# FM-CATH, A Novel Cathelicidin From *Fejervarya Multistriata*, Shows Therapeutic Potential for Treatment of CLP-Induced Sepsis

**DOI:** 10.3389/fphar.2021.731056

**Published:** 2021-08-16

**Authors:** Jiena Wu, Haiyun Zhang, Xiaoxin Chen, Jinwei Chai, Yunrui Hu, Weichen Xiong, Wancheng Lu, Maolin Tian, Xin Chen, Xueqing Xu

**Affiliations:** ^1^ Department of Pulmonary and Critical Care Medicine, Zhujiang Hospital, Southern Medical University, Guangzhou, China; ^2^ Guangdong Provincial Key Laboratory of New Drug Screening, School of Pharmaceutical Sciences, Southern Medical University, Guangzhou, China

**Keywords:** antimicrobial peptide, cathelicidin, sepsis, lipopolysaccharide and lipoteichoic acid-neutralizing, coagulation, cecal ligation and puncture, *F. multistriata*

## Abstract

Sepsis is an exacerbated inflammatory reaction induced by severe infection. As important defensive molecules in innate immunity, several AMPs are reported to prevent septic shock. In this study, we characterized a novel cathelicidin, FM-CATH, from the frog skin of *F. multistriata.* FM-CATH was found to adopt an amphipathic α-helix structural in membrane-mimetic environments and possess favorable antimicrobial effects against bacteria and fungus. In addition, it triggered the agglutination of bacteria. It could also strongly bind to LPS and LTA. Additionally, FM-CATH affected the enzymatic activities of thrombin, plasmin, β-tryptase, and tPA, leading to coagulation inhibition *in vitro* and *in vivo*. Finally, we observed that FM-CATH improved survival rate and inhibited pathological alteration, bacterial count, serum biochemistry, and pro-inflammatory cytokine expression in the cecal ligation and puncture-induced sepsis mice. Taken together, these findings suggest that FM-CATH might be served as a promising agent for the treatment of sepsis.

## Introduction

Sepsis is a syndrome associated with pathological, physiological, and biochemical abnormalities, which is induced by infection and is a life-threatening condition (Huang et al., 2019). Due to the anti-infection effects, the utility of antibiotic therapy in patients with sepsis is widely accepted (Gonçalves de Sousa et al., 2008). However, many antibiotics can stimulate the release of endotoxin and thus increase the occurrence of symptoms (Uppu et al., 2015). Antimicrobial peptide (AMP) is attracting widespread interest as an alternative to traditional antibiotic by augmenting the host response and inhibiting bacterial virulence (Hancock and Sahl, 2006). Lipoteichoic acid (LTA) and lipopolysaccharide (LPS) are important components of the cell wall of many Gram-positive bacteria and the outer membrane of Gram-negative bacteria, respectively (Erridge et al., 2002; Ray et al., 2013), which constitute important virulence factors in bacterial infection and are able to stimulate innate immune responses (Ginsburg, 2002; Heumann and Roger, 2002). LTA and LPS recognize and bind to specific toll-like receptors (TLRs), causing the production and release of many pro-inflammatory mediators from mammal cells (Guha and Mackman, 2001; Rockel and Hartung, 2012). Concurrently, coagulation activated *via* pattern recognition receptors (PRRs) (Ishii et al., 2008; van der Poll and Opal, 2008; van der Poll and Levi, 2012) may promote the development of sepsis (Remick, 2007; Kimbrell et al., 2008; van der Poll and Opal, 2008; Castellheim et al., 2009). Molecules with LPS- and/or LTA-neutralizing activities directly bind to the pro-inflammatory membrane constituents and prevent them from binding to the PRRs (Skovbakke and Franzyk, 2017). Due to their important roles in innate host defense, AMPs have recently received increasing attention in sepsis because they can inhibit proinflammatory responses by directly destroying bacteria or neutralizing LPS or LTA (Vaara and Porro, 1996; Park et al., 2000; Giacometti et al., 2002; Heinbockel et al., 2013; Bosso et al., 2017).

Cathelicidins are one of the largest AMP families which commonly contain a N-terminal signal peptide region, a highly conserved cathelin-like domain and a C-terminal mature peptide (Zanetti et al., 2000). In addition to broad spectrum antimicrobial activity against bacteria, fungi, viruses, and parasites, cathelicidin AMPs also possess diverse biological activities including LPS neutralization, antioxidant, direct chemotaxis, and wound healing effects (Wei et al., 2013; Mu et al., 2017; Cao et al., 2018; Wu et al., 2018). There are abundant studies related to neutralization of LPS or LTA-elicited excessive inflammation during bacterial infection. However, peptides with both LPS and LTA neutralizing activity have been sporadically investigated. In the present study, we characterized a novel cathelicidin from the frog skin of *F. multistriata.* FM-CATH exhibits typical α-helical structure in the membrane mimetic environment and possesses favorable antimicrobial activity, bacterial agglutination activity with high stability and low cytotoxicity to normal mammalian cells. Besides, FM-CATH is able to bind both LPS and LTA, affecting its secondary structure and antimicrobial activity. In addition, our results showed that FM-CATH inhibits coagulation *in vitro* and *in vivo* by affecting the activity of serine protease. Moreover, FM-CATH significantly improves the survival rates and inhibits pathological abnormalities and inflammatory cytokine expression of the CLP-induced septic mice.

## Materials and Methods

### Animals and Ethics Statement

All BALB/c mice (six-week-old) were purchased from the Laboratory Animal Center of Southern Medical University and were housed in the SPF facility at Southern Medical University. The animal experiments were carried out in the light of the approval and guidelines of Animal Care and Use Committee of Southern Medical University. All procedures in this study strictly complied with the Animal Welfare Act and principles stated in the Guide for the Care and Use of Laboratory Animals, National Research Council, 1996.

### Sample Collection, Molecular Cloning, and cDNA Synthesizing

Both sexes of adult *F. multistriata* (n = 3; weighing about 40 g) with no specific permissions in need for sampling were captured from Guangzhou city, Guangdong Province, China (23.12°N, 113.28°E) and were euthanized by CO_2_ before their skins were subsequently sheared and preserved in liquid nitrogen. Total RNA of the *F. multistriata* frog skin was extracted with Trizol (Life Technologies, CA, United States) according to the manufacturer’s protocols and prepared as temples for PCR amplifications as previously reported by us (Chai et al., 2021). The sense primer (5′-GGA​TGA​AGG​TCT​GGC​AGT​GTG​TGC-3′) was used for the 5′ sequence PCR amplifications of cDNA as described in our previous study (Chai et al., 2021). Prediction of physical and chemical parameters of FM-CATH was done by the ExPASy-ProtParam tool (http://web.expasy.org/protparam/).

### Peptide Synthesis

The peptide ordered from GL Biochem Ltd. (Shanghai, China) was purified to 95% with an Inertsil ODS-SP (C18) reverse-phase HPLC column (SHIMAZU, osumi, Japan) before being lyophilized and further confirmed by MALDI-TOF Mass Spectrometry.

### Circular Dichroism Measurement

Circular dichroism (CD) was conducted to identify the secondary structure and the stability of FM-CATH in solutions using Jasco-810 spectropolarimeter (Jasco, Tokyo, Japan) as described in our previous study (Zeng et al., 2018). For secondary structure investigation, peptide at the final concentration of 50 µM were dissolved in 0, 30, 60, 90, and 120 mM SDS solutions. For stability evaluation, FM-CATH (50 µM) dissolved in 60 mM SDS was incubated in 25, 37, 50, 70, and 90°C or 0, 100, 200, and 400 mM NaCl for 1 h before CD spectra measurement. To characterize the binding of peptide to bacteria polysaccharides, 0.2 mg/ml of LPS (L2880, *Escherichia coli* O55:B5, Sigma-Aldrich, St. Louis, Missouri, MO) and LTA (L2512, Staphylococcus aureus, Sigma-Aldrich, St. Louis, MO) were dissolved in H_2_O or 30 mM SDS solution, respectively. FM-CATH was added to the sugar suspension at the final concentration of 50 μM for 1 h at room temperature. Binding of the peptide to LPS and LTA aggregates was studied by monitoring the change in the secondary structure of peptide (Nankar and Pande, 2013). CD data were expressed as the mean residue ellipticity (θ) of three consecutive scans per sample in deg cm^2^·dmol^−1^.

### Antimicrobial Assay

The antimicrobial activity of FM-CATH was measured using two-fold dilution method as reported previously by us (Ye et al., 2020). Microorganisms bought from Guangdong Institute of Microbiology were cultured in Muller-Hinton (MH) broth at 37°C to exponential phase and subsequently diluted with fresh MH broth to reach 10^6^ CFU/ml of density. An equal volume of microbial inoculums in 96-well plates was incubated with different concentrations of FM-CATH at 37°C for 14 h. The absorbance at 600 nm was measured by a microplate spectrophotometer (Infinite M1000 Pro, Tecan Company, Switzerland) to calculate the minimal inhibitory concentration (MIC) values. To measure the inhibitory effect of LPS and LTA on the antimicrobial activity of FM-CATH, LPS or LTA at final concentration of 0.2 mg/ ml was pre-incubation with FM-CATH for 1 h at room temperature before MIC values were measured. Ampicillin and polymyxin B were used as positive controls.

### Stability Analysis

The salt, thermal and serum stabilities of FM-CATH were investigated with antimicrobial assays as described previously by us (Zeng et al., 2018). Briefly, the activities of FM-CATH against *E. coli* ATCC 25922 were measured after peptide incubated with 0, 50, 100, 150, 200, and 400 mM NaCl at room temperature or at 25, 37, 50, 70 and 90°C for 1 h. For serum stability, FM-CATH solubilized in saline was incubated with human serum in a volume ratio of 1:4 for 0–6 h at 37°C before their MICs were determined at 0, 1, 2, 4, and 6 h. All experiments were repeated at least three times.

### Bacterial Agglutination Assay

The agglutination assay was performed with *S. aureus* ATCC 25923 and *E. coli* ATCC 25922. Bacteria at exponential phase were harvested, washed twice and diluted to 10^9^ CFU/ml of density with fresh MH broth. The microorganisms were treated with BSA in TBS, FM-CATH (25 μM), FM-CATH (25 μM) plus equal volume of 0.2 mg/ ml LPS or 0.2 mg/ ml LTA for 30 min at room temperature. The incubation solution was dropped on a glass slide and dyed with a Gram staining kit (Solarbio Technology Co., Ltd., Beijing), and the result was observed under an oil microscope (Nikon Corporation, Japan).

### Cytotoxicity and Hemolytic Assay

The cytotoxicity of FM-CATH on different mammal cells was detected by MTT. Three tumor cell lines (A549, HepG2, and MDA-MB-231) and two normal mammalian cell lines (MH-S and MDCK) were seeded in 96-well plates at a density of 5,000 cells per well. Cells were grown in DMEM or RPMI 1640 medium, or medium containing continuous concentrations of FM-CATH (2.5, 5, 10, 20, 40 μM) at 37°C for 24 h before MTT was added in the dark and the culture was continued for 4 h. The supernatant was removed and DMSO was added before the absorbance at 490 nm was measured. Hemolytic assay was carried out with fresh blood from mouse heart as reported in our previous article with minor modification (Chai et al., 2021). In short, 2% of mouse erythrocyte suspension in TBS solution (v/v) was treated with 2.5, 5, 10, 20, and 40 μM FM-CATH in a 96-V-well plate at room temperature for 2 h. 1% Triton X-100 and PBS were applied as the positive and negative control, respectively. The absorbance of the supernatant at 540 nm was measured with a microplate spectrophotometer. The hemolysis rate was calculated using the following formula: percentage hemolysis = (OD_sample_—OD_PBS_)/(OD_Triton_—OD_PBS_) × 100%.

### Isothermal Titration Calorimetry Assay

ITC assay was carried out to investigate the thermodynamics of interactions between peptides and LPS or LTA using MicroCal PEAQ-ITC (Malvern; United Kingdom). 50 mM of PBS (pH 7.2) was used to prepare stock solutions of peptide, LPS, and LTA. With a constant stirring of 250 rpm/s at 25°C, 1.5 μL aliquots of FM-CATH or polymyxin B at concentration of 1 mM were titrated into the sample cell filled with 280 μL of 50 μM LPS. In LTA binding experiment, 100 μM LTA in the syringe was titrated into 280 μL of 50 μM FM-CATH in the cell with the condition described above. Instrument was operated in high feedback mode. After the heats derived from dilution were subtracted, the equilibrium disassociation constant (*K*_*D*_) and the enthalpy change (ΔH) were analyzed by fitting to a single-site binding model using the MicroCal Origin software. The Gibb’s free energy change (ΔG) and entropy change (ΔS) were obtained from the basic thermodynamic equations, respectively.

### Surface Plasmon Resonance Imaging Measurement

PlexArrayTM HT A100 system (Plexera LLC, Bothell, Washington, United States) and bare gold SPRi chip (Nanocapture gold chip, with a gold layer or 47.5 nm thickness) was used to explore the real-time binding reaction of FM-CATH with LPS, LTA, and proteases as described in our previous method (Chai et al., 2021). In detail, FM-CATH (2 mM) and BSA dissolved in PBS were spotted in multiplex onto the gold chip surface and then stored at 4°C for 14 h in a humid box according to the instruction of the manufacturer. The SPRi chip was washed with PBS and blocked with 1 M ethanolamine/H_2_O solution (pH 8.5) for 30 min, and then mounted in the instrument. Different concentrations of proteinase factors were flowed over the chip at the speed of 2 μL/ s 0.5% (v/v) H_3_PO_4_ in H_2_O was added to regenerate the chip surface. Data were analyzed with the PLEXEA data analysis module and ORIGINLab software (OriginLab).

### Chromogenic Substrate Assay

The chromogenic substrate assay was performed to identify effects of FM-CATH on blood clotting factors. In brief, FM-CATH at final concentrations of 12.5, 25, and 50 μM was mixed with thrombin, plasmin, β-tryptase, tPA for 10 min at room temperature before chromogenic substrates (S2238 for thrombin and β-tryptase; S2302 for plasmin; S2288 for tPA) were added into the mixtures. Substrate hydrolysis at 37°C was measure by reading the absorbance 405 nm at 1-min time intervals with microplate spectrophotometer.

### Anticoagulant Assay *in Vitro*

The anticoagulant activity of FM-CATH was examined by the plasma recalcification time (PRT) and activated partial thromboplastin time (APTT) assays of normal human platelet-free plasma. For PRT measurement, 50 μL of the platelet-poor plasma was mixed with 50 μL of FM-CATH in a 96-well microplate and incubated at 37°C for 10 min before addition of 50 μL of 25 mM CaCl_2_. Absorbance was measured at 405 nm at intervals of 1 min using microplate spectrophotometer. For APTT assay, 90 µL of the platelet-poor plasma was incubated with 10 µL of FM-CATH (final concentration 12.5, 25, and 50 µM) at 37°C for 10 min before 100 µL of pre-warmed APTT assay reagent (Shanghai yuanyeBio-Technology Co., Ltd, China) was added. The 200 µL of mixture was continued to incubate at 37°C for another 5 min before 100 µL of pre-warmed 25 mM CaCl_2_ was added and the clotting time was recorded.

### Bleeding Time Assay

The bleeding time of the transected-tail mice was measured as reported in our previous paper (Ma et al., 2011). Briefly, mice were injected intravenously *via* the tail vein with saline or FM-CATH dissolved in saline (10 mg/ kg). After 1 h, the distal 2 mm segment of the tail was transected and vertically immersed into saline at 37°C. Bleeding time was calculated from bleeding to termination and the end point was the arrest of bleeding lasting for more than 30 s.

### Cecal Ligation and Puncture Sepsis

CLP was carried out as described by us with minor modifications (Chai et al., 2021). In brief, six-week-old BALB/c mice of either gender were randomly grouped to sham, CLP, and FM-CATH-treated groups (n = 6). Each group was weighed and anesthetized with ketamine and xylazine. The cecum was isolated under a sterile environment and ligated at 1.0 cm from its tip before a single puncture was performed in the middle of the ligated cecum with a 20-gauge needle. Subsequently, small amount of feces was extruded and the cecum was returned to the abdomen before the abdomen was sewn up. Sham controls underwent the same surgical procedure but CLP was not performed. Immediately after surgery, percutaneous injection of 1  ml of pre-warmed saline was done into the recovering mice. 2 h after the sham or CLP procedure, mice were intraperitoneally administrated with FM-CATH (10 mg/ kg) or saline, and then the survival rate was recorded every 12 h for up to 3 days. Another set of mice were prepared as described above and the serum, bronchoalveolar lavage fluid (BALF), peritoneal lavage fluid and lung tissues were collected 24 h after the CLP operation for histopathological analysis, pathological scoring, wet/dry weight ratio, bacterial colony forming unit (CFU) counts, cytokine and signaling pathway assays.

### Statistical Analyses

All data were presented as mean ± SEM. Data were analyzed using one-way ANOVA. ^*^ *p* < 0.05, ^**^ *p* < 0.01, and ^***^ *p* < 0.001 were considered statistically significant as compared to control.

## Results

### Identification of FM-CATH

**FIGURE 1:**
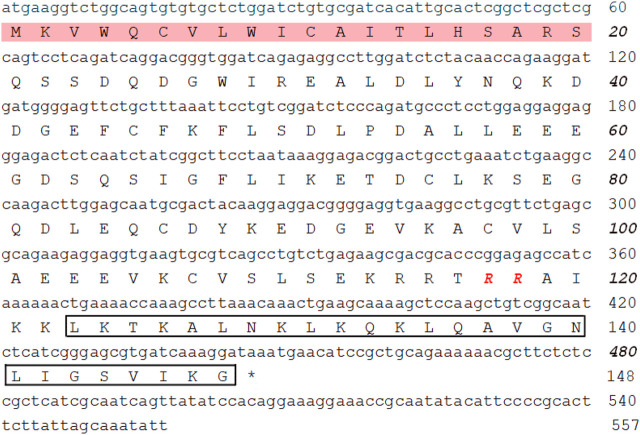
The nucleotide and the deduced amino acid sequences of FM-CATH. The signal peptide is framed in pink and followed by a cathelin-like domain with RR residues at the end in red bole. The sequence of mature FM-CATH is boxed, and the stop codon is displayed by *.

The cDNA encoding one novel antimicrobial peptide named FM-CATH was obtained by PCR-based cDNA cloning method from the cDNA library of *F. multistriata*. The complete nucleotide sequence and translated amino acid sequence of FM-CATH precursor were shown in Figure 1. The cDNA sequence encoding FM-CATH was composed of 557 bp and the deduced amino acid precursor contained 148 amino acids. The sequence of mature FM-CATH was LKTKALNKLKQKLQAVGNLIGSVIKG which displayed high sequence similarities with other representative cathelicidins. FM-CATH had a theoretical PI of 10.70 with + 6 net charge and GRAVY index was -0.004. Its relative mass was measured to 2762.37.

### CD Determination

**FIGURE 2:**
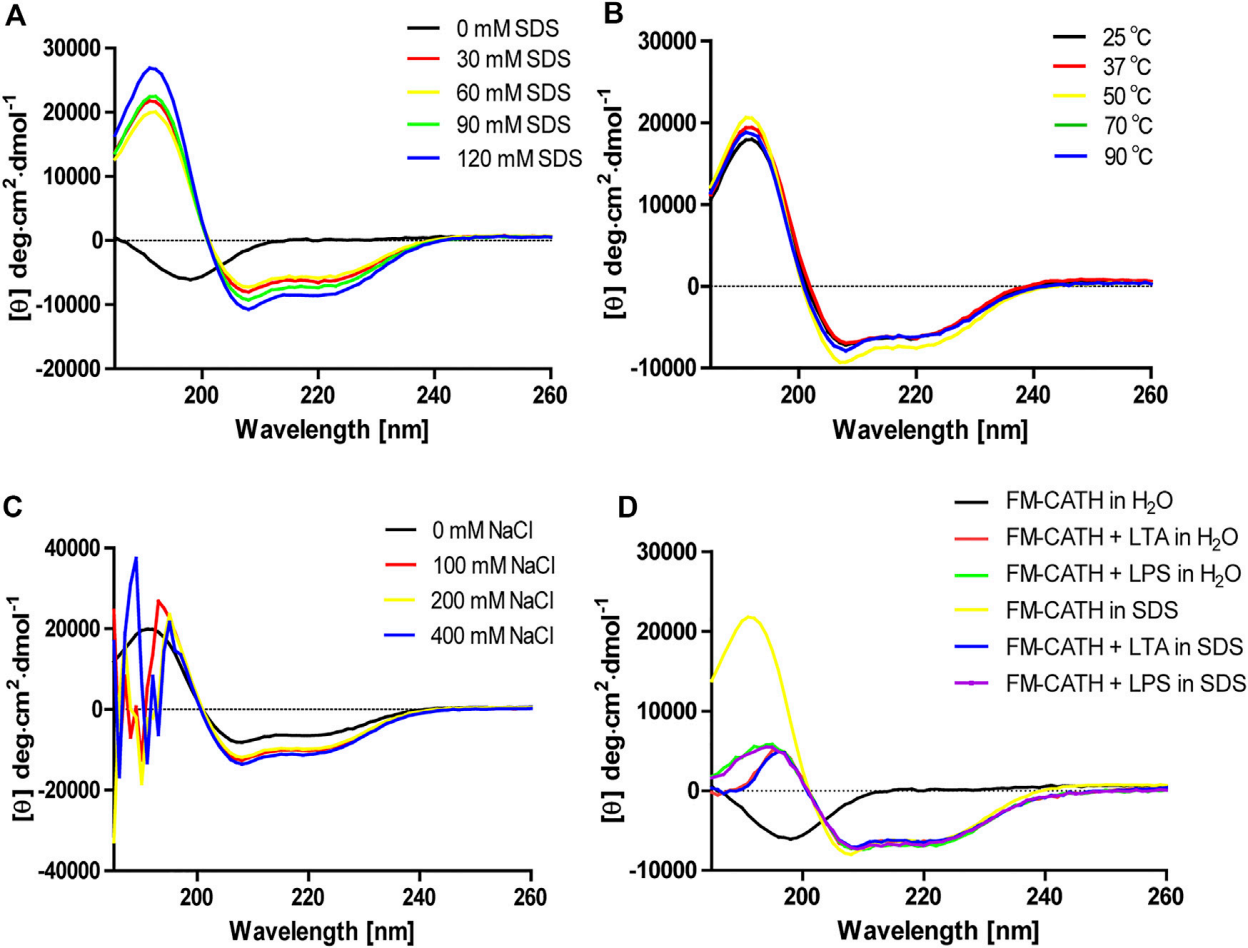
CD analysis of FM-CATH in **(A)** membrane-like SDS solutions, **(B)** 60 mM SDS solution under different temperatures, **(C)** various NaCl concentrations or **(D)** the aqueous solution or 30 mM SDS solution containing LPS or LTA for 1 h before CD spectra measurement.

The secondary structure of FM-CATH as well as its stability in different solution environments were investigated through CD. As presented in Figure 2A, a small negative peak at 198 nm was observed in the CD spectra of H_2_O-dissolved peptide, suggesting that FM-CATH might contain random coil construction. Meanwhile, while dissolved in membrane-like SDS solution, the CD spectra presented a big positive peak at 192 nm and two small negative peaks at 208 and 222 nm, suggesting the presence of α-helix in the secondary structure of FM-CATH. In addition, some slight changes in the secondary structure components of FM-CATH were also observed in different concentration of SDS solution (Table 1). Furthermore, the CD spectra of FM-CATH treated with different temperatures also displayed highly identical peaks (Figure 2B). However, though it showed some changes in various NaCl concentrations (100, 200 and 400 mM), the α-helix structure of FM-CATH was preserved (Figure 2C). Peptides binding to LPS/LTA are known to adopt significant α-helical structures (Nankar and Pande, 2013). To determinate the LPS- and LTA-binding ability of FM-CATH, peptide was individually incubated with the LPS or LTA dissolved in H_2_O or 30 mM SDS solution before the CD spectra were determined, respectively. As presented in Figure 2D, in the presence of LPS or LTA, FM-CATH showed obviously different CD spectra which are features of α-helical structure including two negative bands at 222 and 208 nm and a positive band at 192 nm, suggesting FM-CATH binds to LPS and LTA.

**TABLE 1:** The secondary structure components of FM-CATH in different solution.

Solution	Helix (%)	Parallel (%)	Beta-turn (%)	Random-coil (%)
H_2_O	2.30	42.30	26.00	30.90
SDS (mM)				
30	98.60	1.30	5.10	0.20
60	98.40	1.30	5.50	0.20
90	98.80	1.30	5.20	0.20
120	99.70	0.00	3.80	0.00
NaCl (mM)				
100	92.50	0.70	10.40	0.70
200	99.10	0.10	5.00	0.20
400	99.00	0.20	5.10	0.20
Temperature (°C)				
25	99.20	0.20	3.90	0.30
37	99.30	0.10	3.80	0.20
50	99.40	0.10	3.60	0.20
70	99.30	0.10	3.90	0.20
90	99.50	0.10	3.60	0.20
Bacterial polysaccharides				
FM-CATH + LPS in H_2_O	96.60	0.50	6.70	0.80
FM-CATH + LTA in H_2_O	96.00	0.50	7.70	0.80
FM-CATH + LPS in 30 mM SDS	96.80	0.40	6.90	0.70
FM-CATH + LTA in 30 mM SDS	94.90	0.60	8.30	0.90

### Antimicrobial Activity of FM-CATH

The MICs of FM-CATH against Gram-positive bacteria, Gram-negative bacteria and fungi were presented in Table 2. Under our condition, FM-CATH could suppress the growth of *E. coli* ATCC 25922, *P. acnes* ATCC 6919, *B. subtilis* CMCC 63501, *C. albicans* ATCC 10231, and *P. aeruginosa* ATCC 27853 within a range of 6.25–50 μM, which were more potent or equal to those of polymyxin B. However, its antimicrobial activity against *S. aureus* ATCC 25923 was more than 100 µM. Moreover, the antimicrobial activity of FM-CATH against *E. coli* ATCC 25922 was abrogated after its incubation with LPS and LTA. However, our result showed that the antibacterial activity of polymyxin B at 100 µM was not inhibited in the presence of LPS (0.2 mg/ ml), which is line with the report by Krishnakumari et al. (Krishnakumari et al., 2020).

**TABLE 2:** Antimicrobial activity of FM-CATH.

Microorganisms	MIC (μM)		
	FM-CATH	Ampicillin	Polymyxin B
*Pseudomonas aeruginosa ATCC* 27853	50	>100	50
*Staphylococcus aureus ATCC* 25923	>100	25	>100
*Propionibacterium acnes ATCC* 6919	6.25	>100	6.25
*Bacillus subtilis CMCC* 63501	12.5	>100	25
*Candida albicans ATCC* 10231	6.25	>100	25
*Escherichia coli ATCC* 25922	6.25	12.5	12.5
*Escherichia coli ATCC* 25922**^a^**	>100	12.5	12.5
*Escherichia coli ATCC* 25922**^b^**	>100	12.5	12.5

a Samples were pre-incubated with LPS (0.2 mg/ ml).

b Samples were pre-incubated with LTA (0.2 mg/ ml).

### Stability of FM-CATH

**FIGURE 3:**
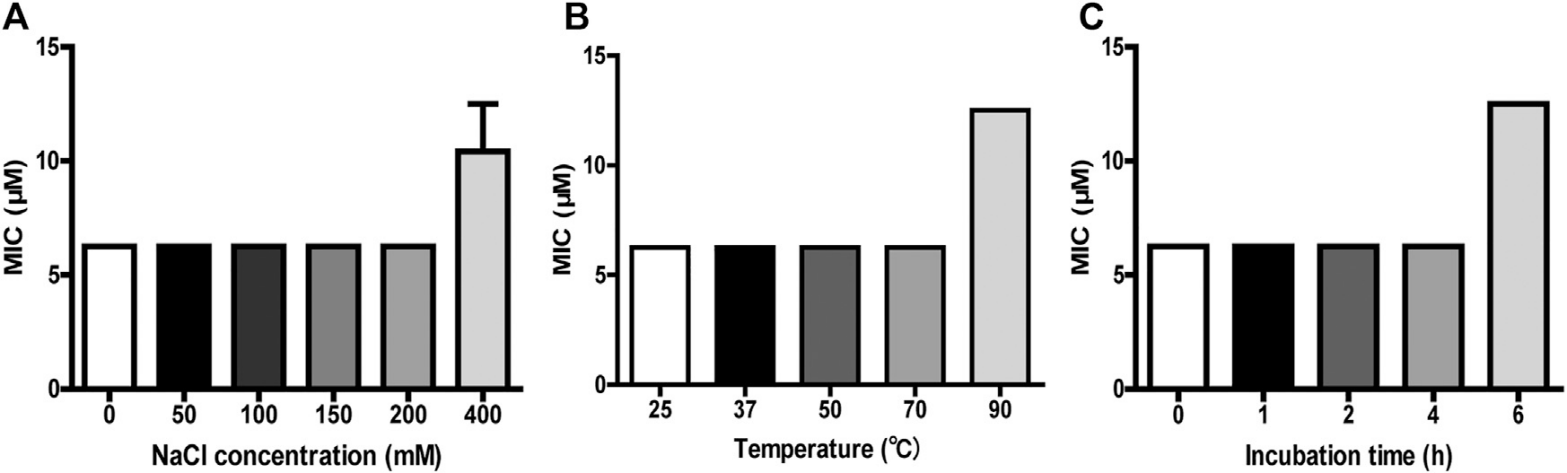
Stability detection of FM-CATH. FM-CATH was incubated **(A)** with different concentrations of NaCl solution at room temperature for 1 h, **(B)** at the indicated temperatures for 1 h, or **(C)** with human serum [1:4 (v/v)] at 37°C for the indicated duration before its MICs against *E. coli* ATCC 25922 were determined. Data are expressed as mean ± SEM (n = 3).

The antimicrobial ability of many AMPs is usually affected by temperature condition, salt concentration, and serum components like proteases (Travis et al., 2000). To further confirm the results from CD experiment, the antimicrobial activity of FM-CATH under different NaCl concentrations and temperature conditions was investigated. As shown in Figure 3, FM-CATH maintained the antibacterial activity against *E. coli* ATCC 25922 at the tested temperatures, NaCl solutions and serum. Although the antibacterial activity decreased after incubation with 400 mM NaCl, solubilization in saline at 90°C for 1 h or human serum (1:4 (v/v)) at 37°C for 6 h, FM-CATH still possessed antibacterial activity. The results indicated that FM-CATH has good stability.

### Interaction Between FM-CATH and LPS or LTA

**FIGURE 4:**
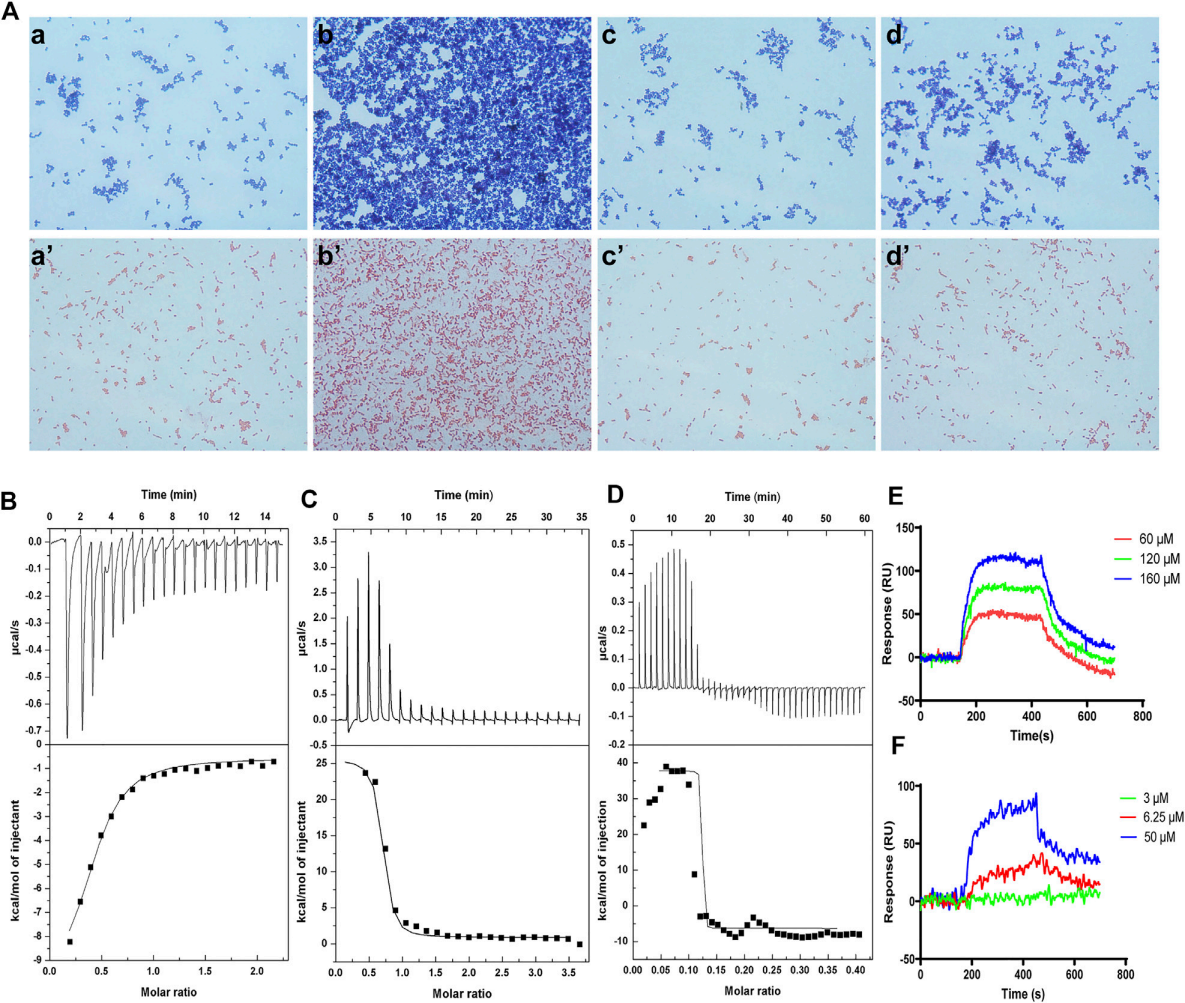
Binding reactions of FM-CATH with LPS and LTA **(A)** Bacterial agglutination of *S. aureus* ATCC 25923 (the upper row) and *E. coli* ATCC 25922 (the lower row), respectively. Bacteria treated with BSA in TBS (a, a’), FM-CATH (25 μM) (b, b’), FM-CATH (25 μM) plus equal volume of 0.2 mg/ ml LPS (c, c’) and 0.2 mg/ ml LTA (d, d’) for 30 min before being stained with Gram staining kit. **(B), (C), (D)** ITC analysis of FM-CATH binding to LPS, polymyxin B binding to LPS, and FM-CATH binding to LTA, respectively. **(E), (F)** SPRi analysis of LPS and LTA binding to FM-CATH immobilized on a gold chip, respectively.

The bacterial agglutination of FM-CATH against *S. aureus* ATCC 25923 and *E. coli* ATCC 25922 was tested. As illustrated in Figure 4A, agglutination occurred in the presence of FM-CATH (25 μM) after 30 min of incubation. However, it was totally abolished by LPS and LTA. ITC experiment was next performed to investigate the binding of FM-CATH to LPS and LTA, respectively (Figures 4B,D). Binding saturation occurred at 12 min while the molar ration of peptide to LPS reach to 20 while the molar ration of LTA to peptide reach to 2at 40 min, respectively. Furtherly, LPS and LTA bound to FM-CATH with *K*_D_ values of about 4.90 μM and 0.714 nM, respectively (Table 3). However, as shown in Figure 4C and Table 3, the *K*_D_ value for polymyxin B binding to LPS was about 0.414 μM, indicating that the binding ability of polymyxin B to LPS was stronger than that of FM-CATH to LPS. SPRi is an optical method monitoring and quantifying biomolecular interactions (Homola, 2008). In agreement, there was strong SPRi signal when LPS and LTA were flowed through the gold chip containing FM-CATH and BSA (Figures 4E,F). Resonance units increased in a concentration-dependent manner and the *K*_D_ values of FM-CATH binding to LPS and LTA were 2.57 μM and 6.21 nM, which was relatively consistent with the ITC results. FM-CATH could thus directly bind to both LPS and LTA.

**TABLE 3:** Thermodynamic parameters from ITC. The parameters (∆H, ∆S, ∆G, *K*_D_) are obtained from the ITC experiments for the interaction of FM-CATH with LPS and LTA plus polymyxin B with LPS, respectively.

Thermodynatic parameters	FM-CATH		Polymyxin B
	LPS	LTA	LPS
∆H (KJ·mol^−1^)	−12.0 ± 0.657	170 ± 5.62	24.7 ± 0.651
T∆S (KJ·mol^−1^)	18.3	222	− 61.2
∆G (KJ·mol^−1^)	− 30.3	− 52.3	− 36.5
*K*_D_ (M)	(4.90 ± 0.495) × 10^–6^	(7.14 ± 2.27) × 10^–10^	(0.414 ± 0.08) × 10^–6^

### Cell Toxicity and Hemolytic Activity of FM-CATH

**FIGURE 5:**
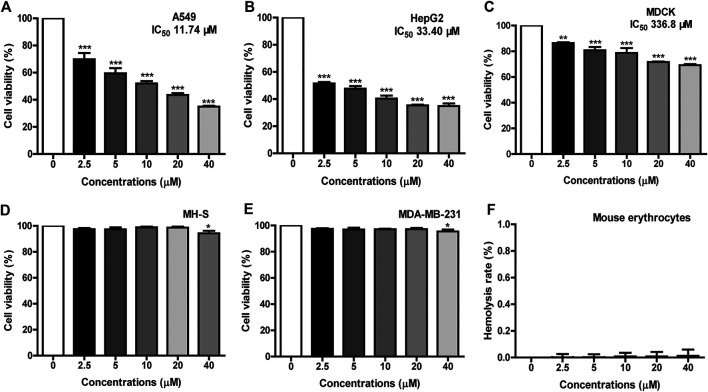
Cytotoxicity and hemolysis of FM-CATH. Cell viability of **(A)** A549, **(B)** HepG2, **(C)** MDCK, **(D)** MH-S, and **(E)** MDA-MB-231 were tested by MTT after incubation with the indicated concentration of FM-CATH for 24 h. The blank medium without any treatment was used as a control. **(F)** Hemolysis rate of mouse erythrocytes with different concentration of FM-CATH. Data are mean ± SEM (n = 3). ^**^ *p* < 0.01 and ^***^ *p* < 0.001 were thought statistically significant as compared to the corresponding control groups.

The application of AMPs is usually limited due to their cytotoxicity to erythrocytes and mammalian cells (Gajski et al., 2016). The cytotoxicity of FM-CATH against different cells were measured by MTT. As illustrated in Figures 5A–E, FM-CATH concentration-dependently inhibited the proliferation of A549, HepG2 and MDCK cells and the IC_50_ values were 11.74, 33.40 and 336.8 μM, respectively. However, it showed very low cytotoxicity against MH-S and MDA-MB-231. Accordingly, we speculated that FM-CATH exerts strong killing activity against some of cancer cells and a low toxicity to normal mammalian cells *in vitro*. Additionally, the hemolytic activity of FM-CATH was measured using mouse heart blood. The hemolysis rates of mouse heart blood at different concentration of FM-CATH were presented in Figure 5F and at the highest concentration, 40 μM, FM-CATH had almost no hemolytic activity.

### Effects of FM-CATH on Coagulation

**FIGURE 6:**
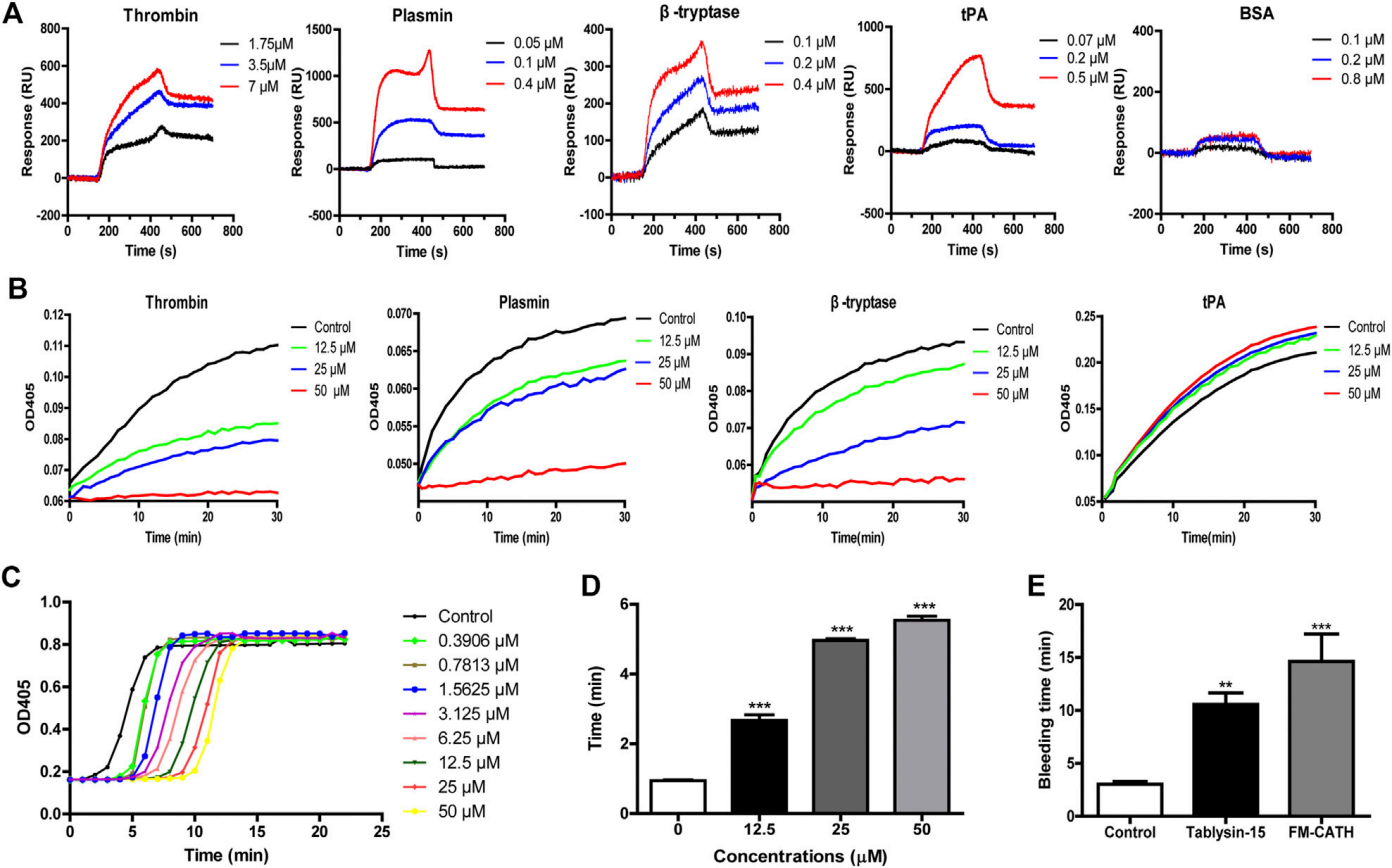
The effects of FM-CATH on coagulation **(A)** SPRi analysis of thrombin, plasmin, β-tryptase, tPA, and BSA binding to FM-CATH immobilized on a gold chip. **(B)** Effects of FM-CATH on chromogenic substrate hydrolysis by serine proteases. The anticoagulation effect of FM-CATH measured by PRT **(C)** and APTT **(D)** assays. **(E)** Effect of FM-CATH on mice tail bleeding time. Data are reported as mean ± SEM (n = 6). ^**^ *p <* 0.01 and ^***^ *p <* 0.001 was considered statistically significant compared with the control group.

To explore whether FM-CATH affected the activities of proteases, its binding reactions were measured by SPRi. As illustrated in Figure 6A, FM-CATH could bind to thrombin, plasmin, β-tryptase, and tPA in a dose-dependent manner but not BSA. In addition, chromogenic substrate assay revealed that FM-CATH increased the activity of tPA while suppressed the one of thrombin, plasmin, β-tryptase (Figure 6B). Given that FM-CATH reacted with serine proteases related to the coagulation cascade, the activity of FM-CATH on plasma was further investigated by PRT and APTT measurements. FM-CATH had ability to prolonged PRT and APTT in a dose-dependent manner, suggesting that coagulation could be inhibited by FM-CATH (Figures 6C,D). To clarify whether FM-CATH had anticoagulant effect *in vivo*, tail bleeding was observed in mice. As shown in Figure 6E, the duration of tail bleeding in the saline-treated group was 3.03 ± 0.52 min. However, the tail bleeding time in the group treated by 10 mg/ kg FM-CATH at a dose of was markedly prolonged to 14.29 ± 4.12 min. The positive control, Tablysin-15, which was described in our previous study (Ma et al., 2011), also significantly extended the tail bleeding time to 10.57 ± 2.67 min at a dose of 2.5 mg/ kg.

### Effects of FM-CATH on CLP-Induced Septic Mice

**FIGURE 7:**
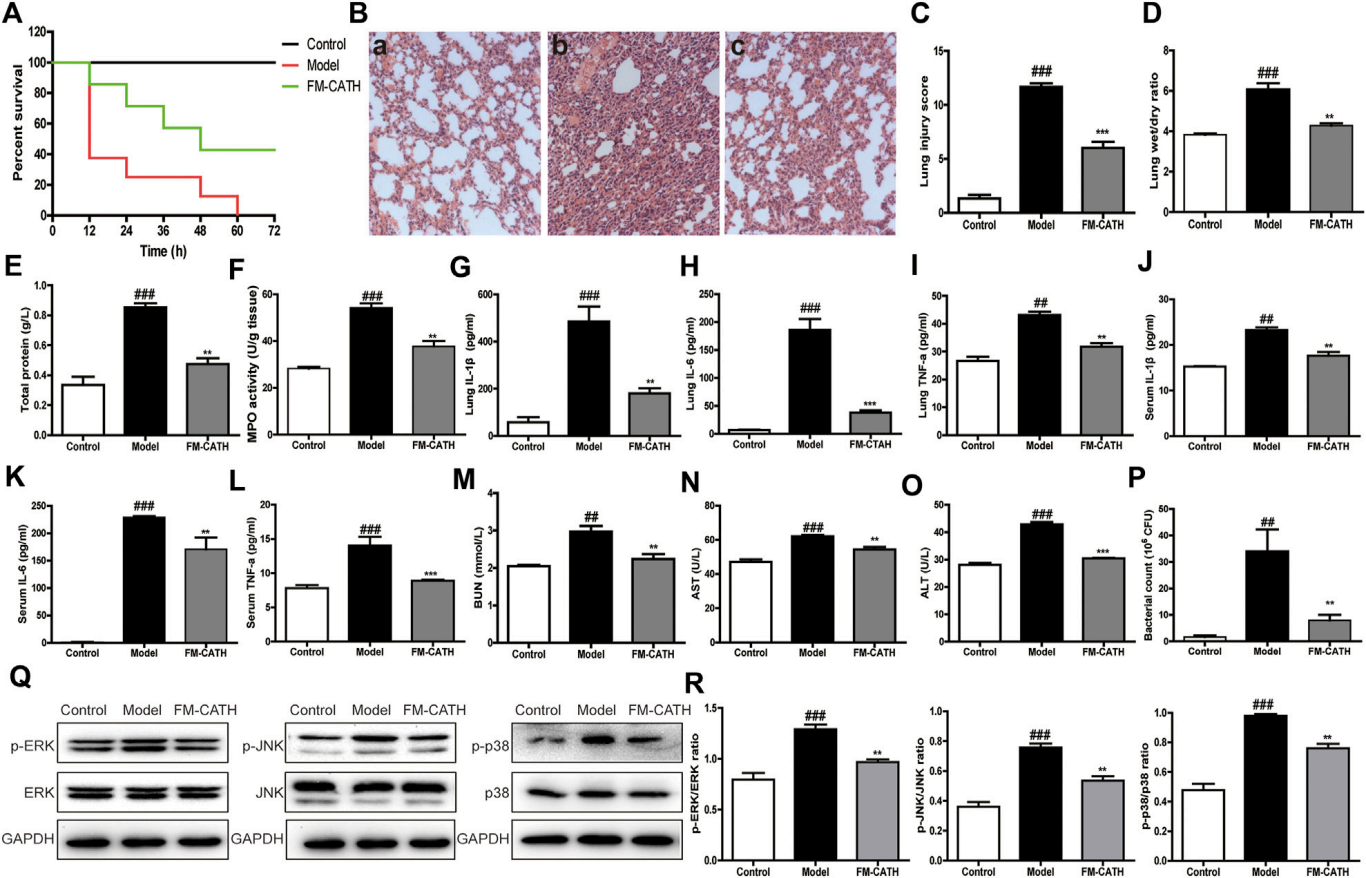
Effects of FM-CATH on CLP-induced mice **(A)** Effects of FM-CATH on the survival rates of CLP-induced mice. **(B)** Representative histological staining images of lung tissues with haematoxylin and eosin. Sham-operated mice were served as negative controls (a), mice were treated with PBS (b), or FM-CATH treatment after CLP induction (c) (magnification × 100) **(C)** Lung injury scores of CLP induction. **(D)** W/D ratio of lung tissues **(E)** The total protein levels of BALF. **(F)** MPO activity of lung **(G–I)** Effects of FM-CATH on pro-inflammatory cytokine expression in lung tissues. **(J–L)** Effects of FM-CATH on pro-inflammatory cytokine expression in serum. **(M–O)** Effects of FM-CATH on BUN, AST, and ALT in serum. **(P)** CFU quantification in the peritoneal lavage fluid **(Q)** Western blotting images of ERK, JNK and p38 expression. **(R)** Ratio of phosphorylated ERK, JNK, and p38 to their total proteins. Data presented are mean ± SEM (n = 5). ^##^ *p* < 0.01, and ^###^ *p* < 0.001 significantly different compared to the control group; ^**^ *p* < 0.01, ^***^ *p* < 0.001 significantly different compared to the model group.

To assess the effect of FM-CATH on polymicrobial sepsis, CLP-induced sepsis mice model was established. Interestingly, FM-CATH obviously improved the prognosis of the septic mice. The survival rate at the assay end point had increased to 43% in the group intraperitoneally injected with FM-CATH while 0% in the control CLP group (Figure 7A). Histological staining of the lung tissue with hematoxylin and eosin in the CLP group showed a severe organ damage including the increase of alveolar wall thickness and inflammatory cell infiltration accompanied by destruction of the alveolar structures and reductions in the alveolar spaces (Figure 7B **,** panel b). It was found that administration of FM-CATH protected against CLP-induced organ damage in the lung (Figure 7B **,** panel c) and lung damage scores were obviously lower than those in the CLP model group (Figure 7C). In line with the above data, the lung wet/dry ratio and BALF total protein level were clearly increased after CLP induction and FM-CATH effectively decreased their levels (Figures 7D,E). Furthermore, MPO activity of lung was evidently abated after treatment with FM-CATH (Figure 7F). Consistently, the FM-CATH treatment also definitely cut down the contents of the inflammatory cytokines IL-1β, IL-6 and TNF-α in the lung tissue (Figures 7G–I) and serum (Figures 7J–L). It was also confirmed that the FM-CATH treatment significantly decreased the contents of some blood biochemical factors like aspartate aminotransferase (AST), urea nitrogen (BUN) as well as alanine aminotransferase (ALT) (Figures 7M–O). Additionally, the FM-CATH treatment reduced the bacterial colony forming unit (CFU) counts in peritoneal fluid (Figure 7P). To further explore the protective mechanism of FM-CATH in CLP-induced sepsis, the expression of MAPK signaling in lung was investigated by western blotting. The activation of ERK, JNK, and p38 were markedly enhanced after CLP administration, which was significantly alleviated by post-treatment with FM-CATH (Figures 7Q,R). Altogether, these data demonstrated that the FM-CATH treatment can significantly increase survival rate, and decrease disease severity in CLP-induced septic mice.

## Discussion

Sepsis is an exacerbated inflammatory reaction induced by severe infection. Thus, it may be more beneficial to inhibit the initial signaling pathways in the infection-related host response. Bacterial products including LPS and LTA, recognized by TLR4 and TLR2, are involved in Gram-negative and -positive bacterial infections, respectively (Mukherjee et al., 2016). Recognition and induction of TLR signaling lead to release of cytokines including IL-1β, IL-6, and TNF-α, which are crucial for the anti-infective immune response (Akira et al., 2006). In addition to direct antibacterial action, several AMPs have been modulators of host responses and prevent inflammation and sepsis (Giacometti et al., 2002; Vonk et al., 2008; Lee et al., 2010). In this study, a cathelicidin is identified from the skin of *F. multistriata* and demonstrated to be a promising new candidate therapeutic for sepsis.

AMPs are relatively small size, cationicity, and amphipathicity in membrane mimetic environment which empower them to damage bacterial membranes selectively (Bosso et al., 2017). FM-CATH possesses a net charge of + 6, indicating its potential to interact with negatively charged bacterial membrane components by electrostatic attraction. Furthermore, like most cathelicidins (Ling et al., 2014; Zeng et al., 2018), FM-CATH adopts an amphipathic α-helix structure in membrane-mimetic environments (Figure 2A). In agreement, FM-CATH possesses a favorable effect against some of bacteria and fungus (Table 2). Additionally, FM-CATH can avoid bacteria moving around and protect the host from pathogenic attack by trapping them together. Though FM-CATH has no exhibit lethal activity against *S. aureus*, its agglutination activity may contribute to prevent the spread of infection (Figures 4A,B).

LPS and LTA play important roles in sepsis and septic shock through hyperactivation of the innate immune system and induction of abnormal coagulation by binding TLRs and PRRs (Guha and Mackman, 2001; Rockel and Hartung, 2012). Our compelling evidences demonstrate that FM-CATH possesses not only direct antibacterial activity, but also direct LPS- and LTA-neutralizing activity (Figure 4). Thus, it is rational to speculate that FM-CATH can effectively control sepsis. Consistently, FM-CATH increases the survival rate, reduces serum biochemistry and the degree of lung injury of CLP mice (Figure 7). Meanwhile, the ability of peritoneal cavity to clear bacteria is enhanced in septic mice. During sepsis, LPS/LTA can activate the MAPK signaling pathway, and further accelerate the production of proinflammatory cytokines, such as IL-1β, IL-6, and TNF-α, which form the cytokine cascade, and eventually leads to cell apoptosis and multiple organ dysfunction (Zhang et al., 2015; Wang et al., 2020). In agreement, the excessive release of inflammatory factors and activation of ERK, JNK, and p38 are inhibited by treatment with FM-CATH.

Coagulation is triggered once plasma tissue factor is induced to express on the surface of monocytes and endothelial cells stimulated by proinflammatory cytokines or bacterial products (Mackman et al., 2007). Therefore, sepsis is almost invariably associated with coagulation abnormalities (van der Poll and Levi, 2012). Surprisingly, our results show that FM-CATH can affect the activity of thrombin, plasmin, β-tryptase and tPA, inhibit plasma coagulation *in vitro*, and prolong the bleeding time *in vivo* (Figure 6). Thrombin is increased during sepsis and plays a role in dysregulated coagulation, and antithrombin is beneficial for patients with severe infection and sepsis in experimental and initial clinical trials (Woodman et al., 2000; Levi and van der Poll, 2017). Furthermore, in sepsis, coagulation abnormalities promote the recruitment of profibrinolytic enzymes including plasmin (Gould et al., 2015). Finally, β-tryptase promotes early pulmonary fibrosis in sepsis-induced lung injury and anti-tryptase treatment with nafamostat mesilate significantly improves the experimental colitis (Isozaki et al., 2006; Villar et al., 2015). Given to the observed anticoagulation effect and protease inhibition/promotion activities of FM-CATH, we cannot exclude that these functions also contributed to its anti-sepsis role *in vivo*.

Alone with the strong and broad-spectrum antibacterial ability, the new AMPs must show low cytotoxicity against human normal cells before their therapeutic use can be considered (Kosikowska and Lesner, 2016). However, as the main target of most AMPs is the cell membrane, part of them also is toxic to host cells, and lead to cytotoxic and haemolytic activity, which are usually major limiting factors for their application (Lee et al., 2014; Kumar et al., 2017). Our results show that FM-CATH exhibits relatively low cytotoxic activities toward the tested normal mammalian cells and low hemolytic activity at the present concentration (Figure 5). In addition to cytotoxicity, stability is also a major reason impeding AMPs from becoming therapeutic agents (Zhu et al., 2015; Wang et al., 2018). As shown in Figure 3, FM-CATH showed better stability in NaCl solution, serum or at different temperature. These characteristics suggest its promising therapeutic potential as anti-microbial drugs.

In conclusion, a novel cathelicidin, FM-CATH, from the skin of frog *F. multistriata* is identified and characterized at present study. FM-CATH contains single α-helix structure in membrane-mimetic environments and favorable antimicrobial, bacterial agglutination, LPS- and LTA-binding activities. In addition, FM-CATH can affect the activities of thrombin, plasmin, β-tryptase and tPA, inhibit plasma coagulation *in vitro*, and prolong the bleeding time *in vivo.* Moreover, FM-CATH significantly protects mice against CLP-induced sepsis. Thus, FM-CATH has the potential for the treatment of sepsis.

## Data Availability

The original contributions presented in the study are included in the article/Supplementary Material, further inquiries can be directed to the corresponding authors.
